# Prevalence and associated factors of treatment failure among HIV/AIDS patients on HAART attending University of Gondar Referral Hospital Northwest Ethiopia

**DOI:** 10.1186/s12865-018-0278-4

**Published:** 2018-12-17

**Authors:** Gizachew Ayele, Belay Tessema, Anteneh Amsalu, Getachew Ferede, Gizachew Yismaw

**Affiliations:** 1 College of Health Sciences, Mizan-Tepi University, P.O. BoX 206, Mizan Teferi, Ethiopia; 2 Departhement of Medical Microbiology college of Medicine and Health Sciences, University of Gondar, Gondar, Ethiopia

**Keywords:** HIV/AIDs, Treatment failure, Immunological failure, Virological failure

## Abstract

**Background:**

The initiation of highly active antiretroviral therapy (HAART) plays a significant role in the clinical management of HIV infected people by preventing morbidity and mortality. This benefit becomes, the most terrible when treatment failure develops. Thus, this research aims to assess the prevalence and associated factors of treatment failure among HIV/AIDS patients on HAART attending University of Gondar Referral Hospital Northwest Ethiopia.

**Results:**

Patients on ART with a minimum of 6 months and up to 12 years of treatment were being enrolled. The prevalence of treatment failure, immunological failure and virological failure among people living with HIV/AIDS attending University of Gondar referral hospital were 20.3, 13.2, and 14.7%, respectively. Patients who had no formal education (Adjusted odds ratio (AOR): 3.8; 95% CI, 1.05–13.77), primary level education (AOR: 4.2; 95% CI, 1.16–15.01) and duration on ART < 6 years (AOR: 2.1; 95%CI, 1.12–3.81) were a significant risk factor. However, initial adult regimen D4T +  3TC+ EFV (AOR: 0.025; 95% CI, 0.002–0.36), AZT +3TC + NVP (AOR: 0.07; 95% CI, 0.01–0.71), AZT +  3TC + EFV (AOR: 0.046; 95% CI, 0.004–0.57) andTDF+3TC + EFV (AOR: 0.04; 95% CI, 0.004–0.46) were significantly protective for treatment failure.

**Conclusions:**

Timely and early identification of associated factors and monitoring antiretroviral therapy treatment failure should be done to enhance the benefit and to prevent further complication of the patients. It is preferable to initiate ART using any one of the following ART regimens: AZT +3TC + NVP, AZT + 3TC + EFV and TDF + 3TC + EFV to prevent treatment failure. Since the prevalence of this treatment failure and its associated factor may be different from other ART centers and community in Ethiopia, further national representative institutional based cross-sectional researches are needed across all ART centers of Ethiopia in order to determine the prevalence of treatment failure and its associated factors.

## Background

Human immunodeficiency virus (HIV) has affected all parts of the world. According to the 2017 UNAIDS report, 36.7 million people were living with HIV in the world. In the same year, one million people died from acquired immunodeficiency syndrome (AIDS) related illnesses and 1.8 million were new HIV infected people [1]. Sub-Saharan Africa is a region highly affected by HIV epidemic. Ethiopia is one of the Sub-Saharan African countries with the highest numbers of people affected by the problem [2, 3]. In Ethiopia, there were 710,000 patients infected with HIV/AIDS in 2016. Around 404,405 HIV patients were on an Antiretroviral therapy (ART) and around 20,000 AIDS-related deaths were reported in the same year [1]. In Amhara regional state, where this research carried out, 191,067 people were living with HIV/AIDS in 2015 [4].

Even though, lack of remedial therapy for HIV/AIDS, the initiation of ART played an important role in the clinical management of peoples infected with of HIV/AIDS [5]. But a significant number of patients fail to achieve a sustained virological and immunological response to treatment among the HIV-infected patients receiving highly active antiretroviral therapy (HAART) [6].

Antiretroviral (ARV) treatment failure is defined as progression of disease and high risk of mortality after beginning of HAART. It can be assessed by clinical failure (occurrence of new infections (OI) or malignancy, symptomatic of clinical disease progression; recurrence of previous OI, onset, or recurrence of WHO stage IV (& certain stage III) conditions, etc.), immunologic failure (a decline in the CD4+ T cell count), or virological failure (The inability to maintain suppression of viral replication to an HIV RNA level < 1000) either in combination or discordantly. Clinical and immunologic criteria have been used for assessing treatment failure in the absence of viral load (VL) test [7–10]. If VL test is available, VL determination and guiding of ART treatment by the gold standard test (VL test) should be recommended [11]. The discordant immune response in patients those doesn’t show a significant increase in the CD4+ T cell count despite viral suppression may also asses with VL and immunological criteria [12, 13].

The patients who had failed ARV therapy treatment are attributed to the higher number of side effects and have the greatest likelihood of experiencing drug resistance and treatment fatigue as a result of being on treatment longer [14]. Development of drug-resistant virus strains (restricting ART alternatives) can be an additional threat if this virus begins to transmit in the population [15, 16]. Therefore, early detection of treatment failure is crucial to sustaining the success of the treatment [17, 18]. Studies conducted in East Africa have revealed that a high prevalence of immunologic failure ranging from 11 to 57% among clients on HAART; moreover, the extent increases as the time of follow-up increases [19–21]. The immunological failure rate in Ethiopia was also found to be high [22].

The timing and accuracy of identifying the risk factors associated with treatment failure include socio-demographic factors (e.g., sex, marital status, educational level, etc.) and clinical factors (e.g., adherence, WHO stage, drug toxicity, ART regimen, CD4+ T cell count, etc.) [23–25] helps to describe timely predictors of treatment efficacy that permit better use of drugs, to avoid unnecessary side effects of ART drug and prevent drug resistance new strain viruses. Identification of risk factors also decrease the economic weight due to a magnificence cost of ART drug and will help as a guide for health professionals and higher officials to alleviate the problem and to develop strategies toward the decreasing rate of treatment failure.

In Ethiopia, viral load determination and guiding ART treatment with the gold standard test (viral load) has started recently because of resource limitation. Consequently, there is limited data on treatment failure and its associated risk factor. The available studies [26, 27] were conducted on first-line antiretroviral treatment failure. These studies didn’t address second-line and immunovirological discordance of treatment failure.

Ethiopia started to implement an ambitious plan launched by UNAIDS in 2014 known as “90–90-90”. This will focus to achieve, at least 73% of all HIV patients will be virally suppressed in 2020 and to end the AIDS epidemic by 2030. The 90–90-90 ambition plan intends to, by 2020; 90% infected individuals with HIV will be diagnosed, 90% diagnosed patients will be receiving ART drug and the viral load in 90% of patients on ART will be undetectable [28]. To achieve this goal, data on immunological and virological treatment failure is required.

Furthermore, the Ethiopia national treatment program needs data to aid the realizing high impact and targeted prevention program; to achive successful elimination of mother to child transmission and improving sustainable quality care and management. Thus, this research was carried out to offer data on prevalence and associated factors of treatment failure among HIV/AIDS patients on HAART attending University of Gondar Referral Hospital Northwest Ethiopia.

## Materials and method

### Study area, design, period and population

A hospital based cross-sectional study and retrospective record review was conducted from February to April 2017. The study was conducted at University of Gondar Referral Hospital which is found at Gondar town, the town is located 747 km from the capital city of the country, Addis Ababa and 182 km far from Bahir Dar which is the capital city of Amhara regional state. According to the recent administration, the town has 12 sub cities which consist of 21 kebeles. Gondar is one of the ancient and densely populated towns in Ethiopia. Based on the figure from the central statistical agency in 2008, Gondar has an estimated population of more than 206,987 (98,085 males and 108,902 females) [29]. In Gondar town, there is one teaching referral hospital providing specialized clinical services, 8 health centers, and 15 private clinics serving the population. Currently 13,753 HIV patients and 5389 on ART patients are attending at the University of Gondar Referral Hospital.

All people living with HIV/AIDS (PLWHIV) and those enrolled in UOG Referral Hospital ART laboratory was the source population. Adult HIV patients who had received ART for more than 6 months, who visit UOG referral hospital ART laboratory during the study period and who consented to be involved in the study was the study population.

All adult people living with HIV and those had had base line CD4 + T cell count, at least 6 months follow up duration of ART and those patients willing to give blood for VL and CD4+ T cell count were included in the study.

Patients who had been seriously sick, unable to give information, incomplete data specially laboratory and clinical data such as:- base line CD4+ T count, base line adherence, base line drug regimen, HIV/AIDS WHO stage, weight, etc., insufficient sample and who were less than 18 years during the study period were excluded from study.

### Operational definition of terms

#### Base line data

The data before ART initiation.

#### Immunological failure

Fall of follow-up CD4 count to baseline (or CD4 falls below baseline), or CD4 levels persisting below 100 cells/mm3, or 50% fall from on-treatment peak value.

#### Virological failure

Plasma viral load greater than 1000 copies/ ml.

#### Responders

Which is sub divided as immunological non-responders (VL >150copies/ml and CD4+ cells increase ≥50/μl) or (VL <150copies/ml and CD4+ cells increase < 50 cells/μl), in comparison with baseline values.

### Adherence

$$ \frac{\mathrm{No}\ \mathrm{of}\ \mathrm{dose}\ \mathrm{of}\ \mathrm{HAART}\ \mathrm{taken}}{\mathrm{No}\ \mathrm{of}\ \mathrm{prescribed}\ \mathrm{dose}\mathrm{s}\ \mathrm{of}\ \mathrm{HAART}}\times 100\% $$

Good adherence, > 95%, fair adherence, 85–95% and poor adherence, < 85% doses take [30, 31].

### Sample size determination and sampling technique

Based on single population formula and systematic random sampling technique with the following assumption, P = population proportion (estimated prevalence) = 0.5, precision, 0.05, by assuming 95% confidence interval α = 0.05 and z(1-a/2) = 1.96d was used for sample size determination. Tree hundred eghity four (*n* = 384) was the minimum sample size. By adding up 10% non-response rate 423 participants were involved.

**Fig. 1:**
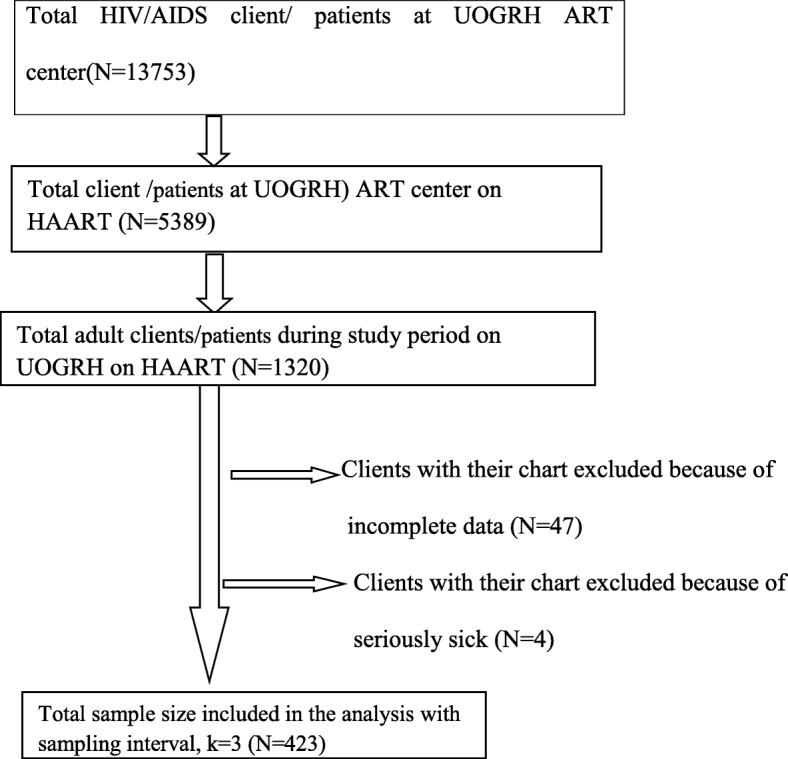
Schematic representation of the sampling procedure of HIV positive adults on HAART at University of Gondar Referral Hospitals from February to April 2017

The average number of HIV/AIDS patients per day under follow up who had given blood for VL and CD4+ T cell count concurrently was 20. During the 3-month data, collection time 1320 HIV/AIDS patients on HAART (> 6 month) were expected to visit the hospital for VL and CD4+ T cell count follow up. Sampling interval (K value) was calculated with 1320/423 = 3.12 = 3. Thus interviews, chart review and blood collection for VL and CD4+ T cell count were conducted at 3 intervals. To determine the first person lottery method was used for the number of patients who had given blood for VL and CD4+ T cell count concurrently at 1st day from the 20 patients. Then each 3rd client was selected for interview, for their chart review and for VL and CD4+ T cell count. If the 3rd patient was absent, clients with their chart were excluded because of incomplete data and seriously sick clients those were excluded because of incapable to give information; the next person was taken as a study subject. The sampling procedure is simplified in the Fig. 1.

### Data collection

#### Socio-demographic data

Socio-demography and potential risk factors data not documented on chart (Alcohol intake, chewing khat, cigarette smoking, etc.) were collected with interview from each study participant using structured questionnaires and relevant clinical features (weight, WHO clinical stage, base line CD4 + T cell count and other consecutive CD4 + T cell count after ART initiation, ART regimen, adherence and duration of HAART, initial regimen, types of co- infections, adverse effect, reason of switching drug) of the patients were retrieved from charts (medical records) by trained nurses working on ART clinic using data extraction checklist. Blood for both CD4 and viral load tests were collected by the laboratory staff at sample collection site.

#### Sample collection and transport

Following standard operational procedure (SOP) sample collection and transport was done**.** About 10 ml venous blood was collected from each patient for CD4 and Viral load tests on the same venipuncture. For both tests 3 -5 ml of whole blood was drawn from each participant using vacutainer tube separately in two tube containing anticoagulant ethylene diamine tetra-acetic acid (EDTA). After centrifugation (3000 rpm for 20 min) plasma was separated and aliquots prepared for viral load testing. Specimens collected were labeled with ART number, study IDs and date and transported directly to the laboratory for CD4+ T cell count and to the molecular laboratory for VL test. The storage temperature for CD4+ T cell count specimen was at room temperature (20 °C – 25 °C) and tested within 24 h. For viral load testing, plasma was separated within 5 h and two aliquots of cryo-vials (with capacity of 1 ml each) prepared for transportation. Specimen transportation was on dry ice and stored at -80 °C until the test is done. Centrifugation, pipetting and aliquoting were performed following standard protocol and laboratory bio-safety precautions both at collection and testing site.

#### Laboratory testing methods

Following SOP quantification of absolute counts of CD4+ T cell on whole blood specimen was done using the FACS Calibur flow cytometer (BD, CA, America) in the laboratory. CD4 + T cells count was done by adding 50 μl whole blood to a reagent tube containing 20 μl of monoclonal antibodies followed by vortexing and incubation for 30 min under dark condition.

Plasma viral load was measured using Quantitative Real-Time PCR HIV-1 assay by the COBAS® AmpliPrep instrument (Roch, Homburg, Germany). Plasma was prepared from 5 ml of blood by centrifuge at 3000 rpm for 20 min.

#### Data processing and analysis

Data were checked every day for its completeness, edited, cleaned and analyzed using SPSS version 21. Analysis was performed using univariate and multivariate logistic regression to determine the extent of the risk factors associated with ART treatment failure. The independent explanatory variable (s) of the dependent variable was selected at univariate analysis and included in multivariate analysis with *p*-value < 0.2. The final association was prepared using multivariate logistic regression. *P*-value < 0.05 was considered significant. After organizing the result in the form of frequencies and percentages, the data were summarized and described using text and tables.

#### Ethical consideration

Ethical clearance was obtained from the University of Gondar, School of Biomedical and Laboratory Sciences Ethical Review Committee and official letter was submitted to the University of Gondar Referral Hospital administration prior to data collection. Written informed consent was obtained from each study participants after explaining the purpose and objective of the study. Patients who were not willing to participate in the study were not forced to participate. All the data and samples obtained from them were kept confidential by using codes instead of any personal identifiers and meant only for the purpose of the study. The laboratory results from the study participants were communicated to their physicians for appropriate management.

## Results

### Socio-demographic characteristics of patients

A total of 423 HIV/AIDS patients who received ART were enrolled in the study. Of these, 272 (64.3%) of them were female and 151 (35.7%) were male. The mean (SD) age of the patients at study time was 39 (+ 9.8) years (range 18–78 years). One hundred sixty-nine (40%) of the patients were within the age group of 30–39 years. The mean weight of patients at the base line and at the time of the study (current) was 50 kg and 56 kg, respectively. At the time of study almost half of patients, 209 (49.4%) were married and 343 (81.1%) were living in urban areas. Three hundred eighty-six (91.6%) were orthodox religion (Table 1).

**Table 1:** Socio-demographic and Clinical characteristics of HIV/AIDs patients in the University of Gondar referral hospital, 2017

Variables		Frequency	Percent
Age	18–29	54	12.8
	30–39	169	40.0
	40–49	136	32.2
	> = 50	64	15.1
Marital status	Single	97	22.9
	Married	209	49.4
	Divorced	81	19.1
	Widowed	36	8.5
Gender	Female	272	64.3
	Male	151	35.7
Resident	Urban	343	81.1
	Rural	80	18.9
Occupation	Farmer	51	12.1
	Merchant	58	13.7
	Student	17	4
	government employ	43	10.2
	daily laborer	62	14.7
	house life	101	23.9
	Private employ	66	15.6
	Other	25	5.9
Educational status	Illiterate	121	28.6
	primary school	117	27.7
	secondary school	142	33.6
	Tertiary	43	10.2
Religion	Orthodox	386	91.3
	Muslim	30	7.1
	Protestant	7	1.7
	Total	423	100.0
Duration of ART in year	<=6	162	38.3
	> 6	261	61.7
Mean ART duration in year	7 year (+ 3)	423	100%
Base line WHO stage	WHO stage I	57	(13.5)
	WHO stage II	97	(22.9)
	WHO stage III	214	(50.6)
	WHO stage IV	55	(13.0)
WHO stage during data collection	WHO stage I	11	(2.6)
	WHO stage II	408	(96.5)
	WHO stage III	4	(.9)
Type of Opportunistic infection	No	308	(72.8)
	Protozoa	4	(.9)
	Helminths	12	(2.8)
	Hepatitis viruses	3	(.7)
	fungal infections	1	(.2)
	TB	89	(21.0)
	Mixed	6	(1.4)
Initial regimen	D4T + 3TC + NVP	78	(18.4)
	D4T + 3TC+ EFV	29	(6.9)
	AZT +3TC + NVP	156	(36.9)
	AZT + 3TC + EFV	30	(7.1)
	TDF + 3TC + EFV	87	(20.6)
	TDF + 3TC + NVP	31	(7.3)
	D4T + 3TC + NVP	6	(1.4)
	Pediatric 4C (AZT + 3TC + NVP)	6	(1.4)
Switching	No	261	(61.7)
	Yes	162	(38.3)
	Total	423	(100.0)
Switching	To 1st line drug	150	(35.5)
	To 2nd line drug	12	(2.8)
Second regimen	AZT +3TC + NVP	61	37.7
	AZT + 3TC + EFV	25	15.4
	TDF + 3TC + NVP	25	15.4
	TDF + 3TC + EFV	39	24.1
	ABC + ddl + LPV/R	11	6.8
	TDF + ddl + IPV/R	1	.6
Reason of switching drug	Toxicity	109	(67.3)
	Pregnancy	7	(4.3)
	TB	18	(11.1)
	Clinical failure	1	(.6)
	Age	9	(5.6)
ARV drug ADH at base line	Good	408	(96.5)
	Fair	2	(.5)
	Poor	13	(3.1)
ARV drug ADH During data collection	Good	420	(99.3)
	Poor	3	(.7)
Base line CD4 count	<=199	267	(63.1)
	200–349	120	(28.4)
	350–499	27	(6.4)
	> = 500	9	(2.1)
CD4 count during data collection	<=199	44	(10.4)
	200–349	110	(26.0)
	350–499	114	(27.0)
	> = 500	155	36.6
Viral load count	Undetected	224	53.0
	0–19	84	19.9
	20–999	53	12.5
	> = 1000	62	14.7
	Total	423	100
Component of immunological failure	CD4 Falling More Than 50%	30	53.6
	CD4 Falling Below Baseline	21	37.5
	CD4 Persistently Below 100	5	8.9
	Total	56	100

*ADH* adherence, *ARV* Antiretroviral, *D4T* Stavudine,*TDF* TenofovirDisoproxilFumarate, *AZT/ 3TC* Zidovudine/Lamivudine, *EFV* Efavirenze, *NVP* Nevirapine, *ABC* abacavir, *ddl* didanosine, *LPV/R* lopinavir/ritonavir, *Mixed* patients infected with more than two organism

### Clinical characteristics of HIV/AIDS patients

The study patients were on ART with a minimum of 6 months up to 12 years with an average time of 7 (± 3) years. Before ART initiation majority of patients had WHO clinical stage III and IV 269 (63.6%), CD4 + T cell count < 200 cells/mm^3^ 267 (63.1%), good adherence 408 (96.5%) and on AZT +3TC + NVP 156 (36.9%) regimen followed by TDF + 3TC + EFV 87 (20.6%).

While at the time of data collection 420 (99.1%) had WHO clinical stage I and II, 44 (10.4%) had CD4 + T count < 200 cells /mm^3^, 420 (99.3%) had good adherence and 162 (38.2%) switched to either first line 150 (35.5%) or second line regimen 12 (2.8%). The most common reason for switching was toxicity 109 (67.3%) followed by TB 18 (11.1%). The common opportunistic infections observed during their ART follow up was TB 89 (21%) (Table 1).

### Treatment failure and associated factors

#### Overall failure

Out of 423 ART patients, 86 (20.3%) had either immunological or virological failure, 56(13.2%) had an immunological failure, 62 (14.7%) virological failure and 20 (7.6%) had both immunological and virological failure and 54 (12.8%) was discordance.

#### Immunological failure and associated factors

Participants were followed for different periods and the total person-time of follow up was 3026 patient-years of follow up. Hence, the rate of immunological failure was 1.85% patient years of follow up. The mean plasma viral load level was 6906 copies/ml (range 0–298,869 copies/ml). Among all the study participants, 56 (13.2%) of patients CD4+ T cell counts during follow-up were below baseline CD4+ T cell count or CD4 levels persisting below 100 cells/mm3, or 50% fall from on-treatment peak value which indicates immunological treatment failure.

In bivariate logistic regression analysis associated factors such as: age of respondent, educational status, duration on ART, initial regimen and viral load during data collection were found to be a *p* value of < 0.2. When it was analyzed with multivariate logistic regression analysis duration of follow up on ART < 6 years, and VL > 20 copies/mm^3^ were significant factors (*p* < 0.0001) for immunologic failure. Patients with a duration follow up on ART < 6 years (AOR = 2.07 (1.11–3.87), *P* = 0.023) and VL > 20 (AOR = 5.2 (2.80–9.62), *P* < 0.0001) were 2 and 5 times more likely to have immunological failure compared with their comparison > 6 years and <  20 copies/mm^3^ respectively (Table 2).

**Table 2:** Bivariate and multivariate analysis of risk factors for immunological failure on ART/AIDs patients attending University of Gondar Referral Hospital 2017

Variables		Immunological failure		COR(95%CI)	AOR(95%CI)	*P* value
		Yes	No			
Age	18–29	13 (24.1%)	41 (75.9%)	2.58 (0.95–7.04)	0.96 (0.25–3.66)	0.955
	30–39	18 (10.7%)	151 (89.3%)	0.97 (0.39–2.45)	0.74 (.26–2.09)	0.570
	40–49	18 (13.2%)	118 (89.3%)	1.23 (0.49–3.14)	1.19 (0.43–3.34)	0.731
	> = 50	7 (10.9%)	57 (89.1%)			Ref
Educational status	Illiterate	18 (14.9%)	103 (85.1%)	2.33 (0.65–8.34)	2.05 (0.53–7.92)	0.297
	primary school	17 (14.5%)	100 (85.5%)	2.27 (0.63–8.16)	2.14 (0.57–0.14)	0.265
	secondary school	18 (12.7%)	124 (87.3%)	1.94 (0.54–6.91)	1.55 (0.41–5.94)	0.522
	Tertiary	3 (7.0%)	40 (93.0%)			Ref
Duration on ART in year	<=6	30 (18.5%)	132 (81.5%)	2.05 (1.16–3.62)	2.07 (1.11–3.87)	0.023^*^
	> 6	26(10%)	235 (90)%			Ref
Initial regimen	D4T + 3TC+ EFV	1 (3.4%)	28 (96.6%)	0.22 (0.03–1.77)	0.29 (0.04–2.49)	0.262
	AZT +3TC + NVP	20 (12.8%)	136 (87.2%)	0.89 (0.41–1.98)	0.73 (0.28–1.92)	0.529
	AZT + 3TC + EFV	2 (6.7%)	28 (93.3%)	0.44 (0.09–2.09)	0.46 (0.09–2.49)	0.370
	TDF + 3TC + EFV	10 (11.5%)	77 (88.5%)	0.79 (0.32–1.98)	0.50 (0.16–1.59)	0.239
	TDF + 3TC + NVP	6(19.4%)	25 (80.6%)	1.46(0.49–4.37)	0.99 (0.29–3.45)	0.996
	D4T + 3TC + NVP	2 (33.3%)	4 (66.7%)	3.04 (0.49–18.67)	1.173 (0.18–7.49)	0.866
	AZT+ 3TC + NVP(4c)	4 (66.7%)	2 (33.3%)	12.18 (1.99–74.67)	12.90 (1.17–142.67)	0.051
	D4T + 3TC + NVP(4a)	11 (14.1%)	67 (85.9%)			Ref
Viral load	> = 20	33(28%)	82(72%)	4.987(2.775–8.963)	5.19 (2.81–9.62)	0.0001^*^
	< 20	23(7%)	285(93%)			Ref

Note:^*^ Has significant association

Those variables with in *p* value < 0.2 under bivariate analysis were included with in multivariate analysis

*COR* crude odds ratio, *AOR* adjusted odds ratio, *Ref* reference, *CI* confidence interval, *p* significant value, *D4T* Stavudine, *TDF* TenofovirDisoproxilFumarate, *AZT/ 3TC* Zidovudine/Lamivudine, *EFV* Efavirenze, *NVP* Nevirapine

#### Virological failure and associated factors

Participants were followed for different period and the total person-time of follow up was 3026 patient-years of follow up. Hence, the rate of virological failure was 2% patient years of follow up. The mean plasma viral load level was 6905.934 copies/ml (range 0–298,869.00 copies/ml). Among all the study participants, 62 (14.7%) of patients were found to have viral load count of > 1000 copies/ml which indicates virological treatment failure.

From bivariate analysis age, marital status, educational status, the reasonfor switching and CD4 count during data collection were had a *p*-value of < 0.2. But multivariate regression analysis showed only CD4 + T cell count during data collection < 200 cells/mm3 and default and age were significant factors (*p* ≤ 0.05) for virological failure. The CD4+ T cell count < 200 cells/mm3 (AOR = 10.09(2.47–41.29), *p* = 0.001) and default and age (AOR = 7.20 (1.99–25.94), *p* = 0.003) was 10 and 7 times more likely to have virological failure compared with comparative group ≥500 cells/mm^3^ and toxicity respectively (Table 3).

**Table 3:** Bivariate and multivariate analysis of associated risk factors of virological failure on ART/AIDs patients attending University of Gondar Referral Hospital 2017

Variables		Virological failure		COR(95%CI)	AOR(95%CI)	*P* value
		Yes	No			
Age	18–29	15 (27.8%)	39 (72.2%)	4.53 (1.52–13.49)	2.18 (0.28–16.98)	0.456
	30–39	22 (13.0%)	147 (87.0%)	1.76 (.63–4.88)	1.15 (0.25–0.45)	0.858
	40–49	20 (14.7%)	116 (85.3%)	2.03(0.72–5.69)	0.91 (0.19–4.44)	0.907
	> = 50	5 (7.8%)	59 (92.2%)			Ref
Marital status	Married	19 (9.1%)	190 (90.9%)	0.32 (.16–.62)	0.61 (0.15-2.44)	0.347
	Divorced	14 (17.3%)	67 (82.7%)	0.67(.32–1.41)	2.52 (0.61-10.45)	0.243
	Widowed	6 (16.7%)	30 (83.3%)	0.64 (.23–1.73)	1.59 (0.27-9.55)	0.665
	Single	23 (23.7%)	74 (76.3%)			Ref
Reason of switching	Tb + pregnant	6 (15.4%)	33 (84.6%)	1.45 (.50–4.18)	1.15 (0.37–3.57)	0.811
	Default + age	8 (53.3%)	7 (46.7%)	9.14 (2.81–29.71)	7.20 (1.99-25.94)	0.003^*^
	Toxicity	12 (11.1%)	96 (88.9%)			Ref
Current CD4 count	<=199	2659.1%	18 (40.9%)	23.43 (9.50–57.77)	10.09 (2.47–41.29)	0.001^*^
	200-349	19 (17.3%)	91 (82.7%)	3.38 (1.46–7.80)	2.94 (0.86–10.07)	0.085
	350–499	8 (7.0%)	106 (93.0%)	1.22 (.45–3.27)	0.83 (0.18-3.80)	0.807
	> = 500	9 (5.8%)	146 (85.3%)			Ref

Note: ^*^Has significant association

Those variables with in *p* value < 0.2 under bivariate analysis were included with in multivariate analysis

*COR* crude odds ratio, *AOR* adjusted odds ratio, *Ref* reference, *CI* confidence interval *p* significant value

#### Discordance

In bivariate analysis age of respondent, duration of ART and reason for switching of the drug had a *p* - value of < 0.2. However, reason for switching of drug (toxicity) were the only significant factors (*p* ≤ 0.05) for discordant with multivariate regression analysis. Switching of drug as a result of toxicity (AOR = 11 (2.16–56.09) *p* = 0.004) was 11 times more likely to cause immune-virological discordance failure than switching of drug as a result of TB, pregnancy default, and age (Table 4).

**Table 4:** Bivariate and multivariate analysis of immuno-virologic discordant on ART/AIDs patients attending University of Gondar Referral Hospital 2017

Variables		Immune-virological failure discordant		COR(95%CI)	AOR(95%CI)	*P* value
		Yes	No			
Age	18–29	10 (52.6%)	9 (47.4%)	0.27(0.04–1.66)	2.46 (0.104–0.04)	0.577
	30–39	22 (71.0%)	9 (29.0%)	0.61(0.10–3.45)	8.29 (0.38–179.94)	0.178
	40–49	14 (53.8%)	12 (46.2%)	0.29(0.05–1.64)	6.51 (0.27–154.25)	0.246
	> = 50	8(80.0%)	2 (20.0%)			Ref
Duration of ART in yrs.	<=6	20 (51.3%)	19 (48.7%)	2.48(1.01–6.09)	5.68 (0.569–56.71)	0.139
	> 6	34 (72.3%)	13(27.7%)			Ref
Reason of switching drug	Toxicity	15 (83.3%)	3 (16.7%)	11(2.15–56.09)	11(2.15–56.09)	0.004^*^
	Others	5 (31.2%)	11 (68.8%)			Ref

Note: ^*^ Has significant association

Those variables with in *p* value < 0.2 under bivariate analysis were included with in multivariate analysis

*COR* crude odds ratio, *AOR* adjusted odds ratio, *Ref* reference, *CI* confidence interval, *p* significant value

#### Associated factors for treatment failure

In a bivariate analysis, patients in the age between 18 and 29 years at the time of data collection were almost 3 times more likely to develop treatment failure (COR: 2.93; 95% CI, 1.22–7.03; *p* = 0.016) as compared to age greater than 50 years. As the educational level increased the treatment failure decreased: patients who have no formal education (COR: 3.64; 95% CI, 0.89–2.33; *p* = 0.043), primary level education (COR: 3.81; 95% CI, 1.09–13.31; *P* = 0.036) and secondary level education (COR: 3.72; 95% CI, 1.07–12.85; *P* = 0.038) were 3.6 times, 3.8times and 3.7 times, respectively as compared to those who have tertiary level education. However, patient had initial HAART regimen of D4T + 3TC + NVP (COR: 0.075: 95% CI, 0.01–0.71; *P* = 0.0240), D4T + 3TC + EFV (COR:0.02; 95% CI, 0.001–0.25; *P* = 0.003), AZT +3TC + NVP (COR: 0.06; 95% CI, 0.01–0.57; *p* = 0.014), AZT + 3TC + EFV (COR: 0.04; 95% CI, 0.003–0.44; *p* = 0.009), TDF + 3TC + EFV (COR: 0.05; 95% CI, 0.01–0.46; *p* = 0.009) were significantly protected from treatment failure as compared to patients who are on AZT + 3TC + NVP of child regimen. In a multivariate logistic regression analysis, only patients who had no formal education (AOR: 3.8; 95% CI, 1.05–13.77; *p* = 0.042), primary level education (AOR: 4.2; 95% CI, 1.16–15.01; *p* = 0.029) and duration on ART < 6 years (AOR: 2.1; 95% CI, 1.12–3.81; *p* = 0.021) were a significant risk factor. (However, initial adult regimen D4T + 3TC+ EFV (AOR: 0.025; 95% CI, 0.002–0.36; *p* = 0.007), AZT +3TC + NVP (AOR: 0.07; 95% CI, 0.01–0.71; *p* = 0.025), AZT + 3TC + EFV (AOR: 0.046; 95% CI, 0.004–0.57; *p* = 0.016) and TDF + 3TC + EFV (AOR: 0.04; 95% CI, 0.004–0.46; *p* = 0.009) were significantly protective for treatment failure (Table 5).

**Table 5:** Bivariate vs multivariate analysis of treatment failure on adult HIV/AIDS patients attending University of Gondar Referral Hospital 2017

Variables		Treatment failure		COR (95%CI)	AOR(95%CI)	*P* value
		Yes	No			
Age	18–29	19(35%)	35(65%)	2.93 (1.22–7.04)	1.59 (0.54–4.72)	0.398
	30–39	31(18%)	138(82%)	1.21(0.56–2.64)	1.13 (0.49–2.61)	0.772
	40–49	26(19%)	110(81%)	1.28(0.57–2.84)	1.31 (0.56–3.04)	0.530
	> = 50	10(16%)	54(84%)	Ref		Ref
Educational status	No formal education	26(22%)	95(79%)	3.65 (1.05–12.75)	3.89(1.10–13.77)	.035^*^
	primary school	26 (22%)	91(78%)	3.81 (1.09–13.32)	3.730(1.06–13.18)	.041^*^
	secondary school	31 (22%)	111(78%)	3.72 (1.08–12.86)	2.93(0.83–10.38)	.096
	Tertiary	3 (7%)	40(93%)	Ref		
Duration of ART in yrs.	< 6	39(24%)	123(76%)	1.44 (0.89–2.33)	2.1 (1.12–3.81)	0.021^*^
	6	47(18%)	214(82%)			Ref
Initial regimen	D4T + 3TC + NVP	18 (23%)	60(77%)	0.08 (0.01–0.71)	0.10 (0.01–1.06)	0.056
	D4T + 3TC+ EFV	2 (7%)	27(93%)	0.02 (0.001–0.25)	0.03 (0.002–0.37)	0.007^*^
	AZT +3TC + NVP	31 (20%)	125(80%)	0.06 (0.01–0.57)	0.07 (0.01–0.72)	0.025^*^
	AZT + 3TC + EFV	4(13%)	26(87%)	0.04 (0.003–0.44)	0.05 (0.004–0.57)	0.016^*^
	TDF + 3TC + EFV	14(16%)	73(84%)	0.05 (0.01–0.46)	0.04 (0.004–0.46)	0.009^*^
	TDF + 3TC + NVP	9 (29%)	22(71%)	0.102 (0.01–1.045)	0.11 (0.01–1.25)	0.075
	D4T + 3TC + NVP(4a) child regimen	4 (57%)	3(43%)	0.33 (0.02–4.736)	0.32 (0.02–4.76)	0.408
	AZT + 3TC + NVP(4c) child regimen	4 (80%)	1(20%)			Ref
	AZT +3TC + NVP	18(30%)	43(71%)	Ref		

Note: ^*^ Has significant association

Those variables with in p value < 0.2 under bivariate analysis were included with in multivariate analysis

*COR* crude odds ratio, *AOR* adjusted odds ratio, *Ref* reference, *CI* confidence interval, *p* significant value, *D4T* Stavudine, *TDF* TenofovirDisoproxilFumarate, *AZT/ 3TC* Zidovudine/Lamivudine, *EFV* Efavirenze, *NVP* Nevirapined, *di* Didanosine

## Discussion

Identifying and managing treatment failure are a basic challenge for national treatment program. Sustainable treatment failure is related to difficulty to delivering quality care, the emergence of drug resistant viruses which limits the treatment option and increases the threat of morbidity and mortality. Thus the study was intended to assess the prevalence and associated factors of treatment failure among HIV/AIDS patients on HAART.

The prevalence of treatment failure as a result of immunological failure 56 (13.2%) identified in this study was similar to those reported by other studies done in Bahir Dar [27] and SNNP region, Ethiopia [22] and Uganda [19] showing an immunological failure of 15.9, 11.5 and 11% respectively. However; our result was slightly lower than the study done in Bahir Dar Ethiopia. The difference might be as a result of the fact that the clients (patients) of the study participated in Bahir Dar were only under the first line HAART regimens, but our study patients were either under first or second- line of HAART regimen. Since most failures occur soon after the switch is made from the first line to a second-line therapy, a shorter follow-up period is most likely to find a higher probability of failure when compared to a study with a longer follow-up period.

Our finding showed that the duration of follow up on ART < 6 years (*P* = 0.023) was significantly associated with immunological failure and it was 2 times more likely to cause immunological failure than the duration of follow up on ART > 6 years. This finding was inconsistent with a study done in French [32]. The discrepancy might be due to lack of assessment of new strain ART drug resistance viruses and its prevalence rate. If the duration of follow up on ART increases (late after the introduction of HAART drug in 2005 in Ethiopia), the emergence of some new HIV drug resistance (HIVDR) strain is predictable as a result of HIV’s error-prone replication, mutation rate and viral recombination. If this new strain HIVDR virus infects others, treatment failure may be occurring and management may not be effective. So patients with short duration of follow up (< 6 year) on HAART has had two times the chance of acquiring the new strain drug resistant virus either during the duration of treatment follow up or initially by transmission than those patients infected with HIV (less likely drug resistant virus) before or early the introduction of ART even though those have had long duration of follow up (> 6 year) on ART to acquire this new strain drug resistant virus. So the high prevalence of drug resistant virus with immunological failure through reduction of CD4+ T cell count is expected in patients those ages is < 6 years than those have had > 6 years of follow up on ART.

Viral load ≥20 (*P* = 0.001) was the other significant factor for immunological treatment failure. This finding was consistent with other studies done in South Africa [33] and Eurosids [34] those showed viral load had the greatest impact on the CD4+ T cell decline. For patients with high viral load (more viruses) may trap more CD4+ cells in lymphoid tissue, which results in a low CD4+ cell count in blood [27].

The prevalence of treatment failure due to virological failure in our research was 14.7% (64/423) among the study participants. This finding was in agreement with a research done in Zambia [35] (11.7%), Tanzania 12.3% [36], Malawi 9.2% [37], Nigeria 13.7% [38], South Africa 13.7% [39] and Uganda 11.3% [40]. However; our finding was lower than the other research done in Cameron 23.2 [41] and coastal Kenya 24% [42] and slightly higher than the study conducted Uganda 9.9% [19], Bahir Dar 10.7% [27] and Gondar 4.1% [26]. The observed variation may be as a aresult of difference in geographical area, the study design, the endpoint of virological failure and sample size.

Our finding showed that CD4 + T cell count during data collection < 200 cells/mm3 (AOR =10.09 (2.47–41.29), *p* = 0.001) were 10 times more likely to have virological failure contrasted with comparative group ≥500 cells/mm^3^ it was in line with the research done in Peru [43] and Kenya [44]. Since the diminishing of HIV-1-specific CD4 T helper cell, as a result of decline in CD4 T cell counts, qualitative impairments of CD4 T cell function and functional impairment of CD4 T cell indirectly lead to a functional impairment of HIV-1specific CD8 T cells [45] or B cell responses to control viral replication [46]. So the amount of viruses in the body increased.

In this research reason of switching as a result of default and age (AOR = 7.20 (1.99–25.94), *p* = 0.003) was 7 times more likely to have virological failure contrasted with the comparative group toxicity. It is inconsistent with studies done in Uganda [47] and Nigeria [48] those showed TB, breastfeeding, and pregnancy was significant for virological non-suppressed. This difference might be due to 9/15 (60%) of switching as a result of default and age in our study were within the age range of 18–29, with 53.3 virological failures. Those aged < 30 years at the time of starting ART were more likely to break off the regimen [49] results in a significant rise of viral load.

Antiretroviral therapy treatment failure with immune-virologic discordance in our research was 54 (13%). This finding was concordant with other research done in India that showed 28 (13.6%) discordant patients [50]. However, a research carried out in Nigeria [45] showed 33% immune-virologic discordance was inconsistent with our finding. This disagreement is most likely due to the difference in sample size (206 study subject) and study design (cohort study) that applied in Nigeria.

Our findings showed that only switching of drugs as a result of toxicity (AOR = 11 (2.15–56.09), *p* = 0.004) was significant factors for discordant results it is in line with other studies done from the computerized database retrospectively [51, 52]. The potential causes might be marrow-suppressive medications and infiltrative bone marrow processes as a result of toxicity of known marrow suppressive drugs used in HIV-infected patients comprise zidovudine (Retrovir) and zidovudine-containing fixed combination pills that effect poor CD4 cell count responses in the setting of persistent virologic suppression and Toxicity as a result of TDF that causes the occurrence of bone fractures or changes in fat distribution this may also affect CD4 lineage [53].

Our study showed that the educational level was significantly associated with treatment failure. This finding was inconsistent with the research done in Addis Ababa, Ethiopia [54] and Bahir Dar, Ethiopia [27]. The difference may be due to the study participant awareness for treatment failure, for instance, the study participant in Addis Ababa were all from Urban but our study subject was comprised 79% urban and 21% rural with 23% no formal educational level.

Even though the regimen containing AZT + NVP can induce high incidence of the bone marrow suppression with encouraging high rate of lukimia, the goals of ART are actually the qualitative and quantitative immunological reconstitution and the maximum possible reduction of the viral load in the longest possible time [1, 55]. In line with that, the results of this research showed that initial adult regimens such as D4T + 3TC+ EFV (*P* = 0.007), AZT +3TC + NVP (*P* = 0.025), AZT + 3TC + EFV (*P* = 0.016), TDF + 3TC + EFV (*p* = 0.009) were significantly protective for treatment failure as compared to regimen AZT + 3TC + NVP. This finding was in line with the study done in Ethiopia [56].

Duration of follow up on ART ≤6 years (*p* = 0.021) was significantly associated with treatment failure and was 2 times more likely to cause treatment failure than the duration of follow up on ART > 6 years this is in line with the study conducted in the Malaysian state of Selangor [57]. This finding was not in line with research done in Bahir Dar, Ethiopia [22] Kumasi, Ghana [58]. This is most probably due to the lake of initial viral load measurement and increment of the transmission of the drug-resistant viruses.

### Limitations

Acquired drug resistance testing was not done, because the testing facility has not been so far available in the country. The patient’s clinical data was taken from the records retrospectively. Excluding patients who were seriously sick, since unable to get data during interview and lack of sufficient blood. The virological failure rates were assessed based on a single point testing of viral load, so there may be missed classification of HIV treatment failure. The prevalence of treatment failure is not really a populated-based estimate and is not representative of the broader population of people who are on ART and at risk of treatment failure. Despite these limitations, this study provides important information which would be useful for the ART treatment programs in the country**.**

## Conclusion

The prevalence of treatment failure, immunological failure and virological failure among ART patients attending UOG referral hospital were 20.3, 13.2, and 14.7% respectively. Fifty-four (13%) patients were discordant. Even though no formal education, primary level education and duration on ART < 6 years were a significant risk factor of treatment failure, initial adult regimen D4T + 3TC+ EFV, AZT +3TC + NVP, AZT + 3TC + EFV, and TDF + 3TC + EFV were significantly protective. One of the following ART regimens: AZT +3TC + NVP, AZT + 3TC + EFV and TDF + 3TC + EFVare recommended. Switching as a result of toxicity was significant risk factor of immuno-virological discordance. Since it is single ART center study, the result may not also be generalized to all hospitals, so further study is needed to be done in a wider community and multiple ART centers to determine whether there are differences in virological and immunological responses and immune-virological discordance to antiretroviral therapy at different stages of HIV infection.
